# A POLICY- AND PRACTICE-ALIGNED RESEARCH AGENDA FOR ENGAGING AND SUPPORTING DEMENTIA CAREGIVERS IN CARE DELIVERY

**DOI:** 10.1093/geroni/igac059.283

**Published:** 2022-12-20

**Authors:** Catherine Riffin, Francesca Falzarano, Sara Czaja

## Abstract

The Cornell Institute for Translational Research on Aging (CITRA) Research-to-Practice Consensus Workshop Model is an evidence-based method for generating practice- and policy-informed research agendas on aging-related topics. The model aims to bridge the gap between research-based knowledge and practice-based insight by involving multidisciplinary stakeholders in all aspects of the agenda-setting process. Using the CITRA model as a guiding framework, we convened an NIA-funded Conference on Engaging Family Caregivers of Persons with Dementia in Healthcare Delivery, with the goal of generating actionable research priorities that will lead to improvements in dementia caregiver identification, assessment, and support in health and long-term care settings. Conference attendees were multidisciplinary thought leaders representing five stakeholder groups: family caregivers of persons with dementia, healthcare providers, researchers, payers, and policy advocates. This symposium will describe the CITRA model, using our caregiver conference as an example, and provide practical guidance on how to use the model to promote cross-disciplinary dialogue and integrate research, policy, and practice perspectives. The first presentation will provide an overview of the CITRA model and its 5-step method (Pillemer). Subsequent presentations will describe the model’s application to the topic of improving caregiver identification and support in care delivery (Griffin) and discuss how novel technology-based adaptations to the model helped to facilitate hybrid participation of virtual and in-person attendees (Falzarano). The final presentation will delineate the major priorities that resulted from the conference, discuss ongoing and future dissemination activities, and offer practical suggestions for leveraging the CITRA model in future consensus efforts (Riffin).

